# Orthodontic Elastic Embedded in Gingiva for 7 Years

**DOI:** 10.1155/2013/212106

**Published:** 2013-07-30

**Authors:** Shruti Tandon, Abdul Ahad, Arundeep Kaur, Farrukh Faraz, Zainab Chaudhary

**Affiliations:** ^1^Department of Periodontics and Oral Implantology, Maulana Azad Institute of Dental Sciences, Bahadur Shah Zafar Marg, New Delhi 110002, India; ^2^Department of Oral and Maxillofacial Surgery, Maulana Azad Institute of Dental Sciences, Bahadur Shah Zafar Marg, New Delhi 110002, India

## Abstract

Dental materials especially orthodontic elastics often get embedded in gingival tissues due to iatrogenic factors. If retained for a long time, inflammatory response starts as asymptomatic crestal bone loss and may progress to severe periodontal abscess. Unsupported orthodontic elastics used for diastema closure may result in exfoliation of teeth, while elastic separators may get embedded in interdental gingiva if banding is performed without removing it. These cases of negligence are detrimental for survival of affected teeth. This paper highlights a case of orthodontic elastic embedded in interproximal gingiva of a 23-year-old healthy female for 7 years after completion of fixed orthodontic treatment. Surprisingly, there was no clinical sign of inflammation around elastic band and it was removed easily without any local anaesthesia. However, mild crestal bone loss was observed on periapical radiograph. The gingiva healed completely after sub gingival debridement.

## 1. Introduction

The presence of foreign bodies in gingiva, leading to inflammatory response, is unusual but not a rare condition. Most of the cases in the literature have been reported to be iatrogenic, commonly associated with use of elastic bands and separators for orthodontic treatment [1–3]. Other dental materials like amalgam, composite, cements, and prophylaxis paste have also been found to be embedded in gingiva [4]. The resulting inflammatory response varies from asymptomatic mild crestal bone loss to severe periodontal destruction causing abscess formation [5, 6].

Most of the cases in literature have been reported to be most common in mandibular posterior region (34%), followed by maxillary posterior (29%) and maxillary anterior regions (26%). Probably this incidence correlates with more dental treatments received in these regions [7].

Unsupported orthodontic elastics creeping into gingival sulcus have been reported frequently in the literature [8–10]. Some authors have also reported the presence of elastic separators in interproximal area that are used for relieving contact before band placement [5, 6, 11–13]. This report describes a case of intact orthodontic elastic found embedded in interproximal gingiva between mandibular first and second molars, 7 years after completion of orthodontic treatment.

## 2. Case Presentation

**Figure 1:**
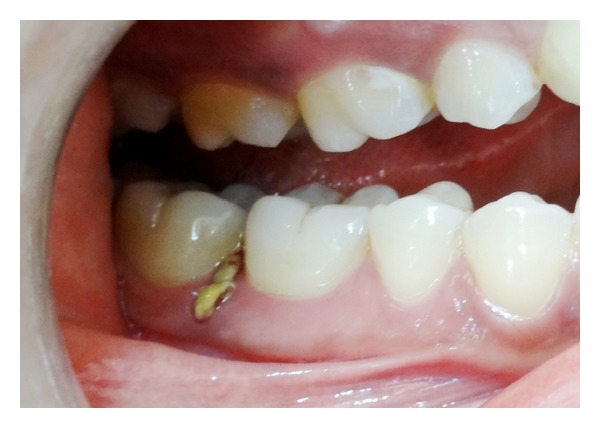
Elastic band protruding through interdental gingiva between right mandibular first and second molars.

**Figure 2:**
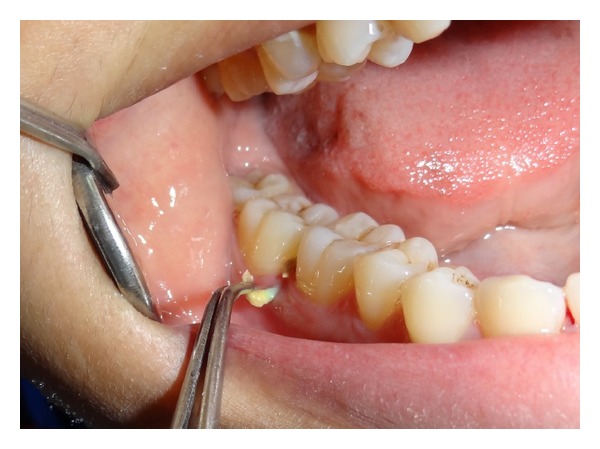
Elastic band was easily removed using forceps.

**Figure 3:**
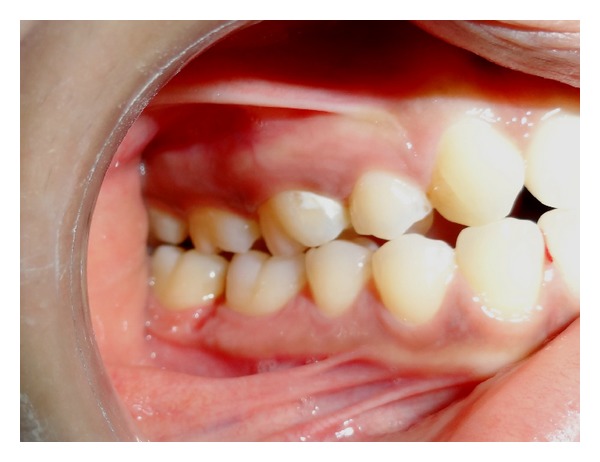
Indentation of elastic band on interdental papilla after its removal.

**Figure 4:**
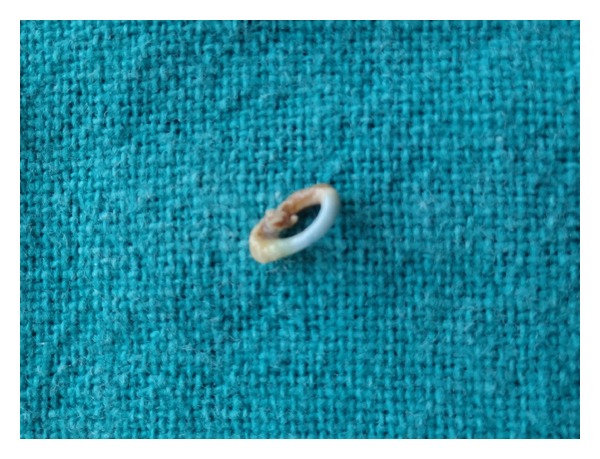
Intact elastic band. Half of the band was covered with heavy plaque while the other half was relatively cleaner.

**Figure 5:**
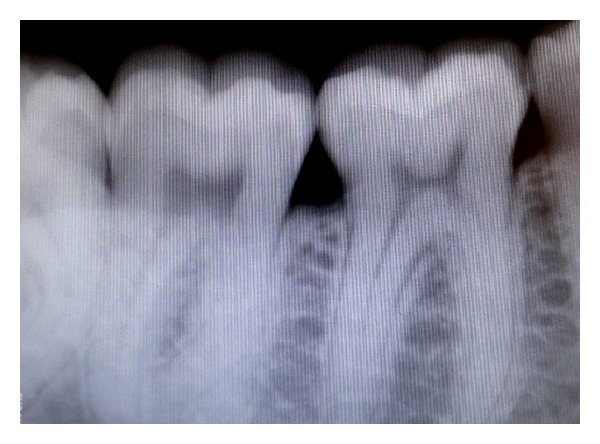
Periapical radiograph showing mild crestal bone loss between first and second molars.

**Figure 6:**
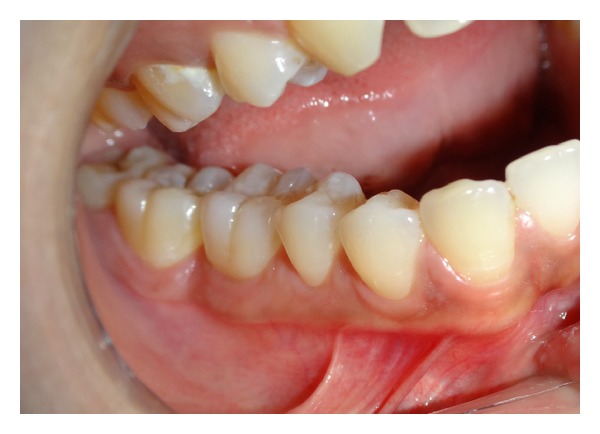
Completely healed gingiva after 1 month.

A 23-year-old female reported for routine oral prophylaxis. She complained of occasional bleeding from gums on brushing. There was no history of pain; however she reported to have noticed a yellow growth on gingiva between right mandibular posterior teeth, for last 1 month. The medical history was not significant. The patient had completed fixed orthodontic treatment for crowded anterior teeth when she was 16 years old. On examination, oral hygiene was found to be fair except mild deposits of calculus. As reported by the patient, a yellow coloured material was found protruding through interdental papilla between right mandibular first and second molars (Figure 1). When held with forceps, an intact elastic band came out easily, without bleeding or any discomfort to the patient (Figure 2). An indentation of the elastic band was found on the buccal aspect of interdental papilla (Figure 3). Heavy plaque was present on the part of the elastic band that was protruding out while the other part was relatively cleaner (Figure 4). To rule out any other foreign bodies, an IOPA radiograph was taken that revealed only mild crestal bone loss (Figure 5). The area was debrided using Gracey curettes nos. 11-12 and nos. 13-14 (Hu-Friedy, Chicago, IL, USA) and irrigated with normal saline. Patient was advised to do warm saline rinses 3 times daily for 1 week. Patient was recalled after 1 week, and she reported no incidence of pain or any discomfort in the area. After 1 month, there was complete healing of gingiva (Figure 6).

## 3. Discussion

Elastic bands are commonly used in orthodontics for space closure, derotation, correction of cross bite, and as separator before band placement. Ideally, elastics other than separators should be stabilized by bonded attachments or brackets and evaluated at regular intervals. It is recommended that under no circumstances any unsupported elastic be looped around teeth for diastema closure [8]. However, this is still in practice by clinicians and due to negligence or failure to follow up, this often results in creeping of elastic band apically into gingival sulcus, along the root surface [9]. In the case reported here, it was unusual that the patient was oblivious of the elastic embedded in the gingiva for the last 7 years, although a part of it was protruding out of the interdental papilla. The gingiva had grown around the elastic in a tunnel-like manner, such that pulling it out with forceps did not require local anaesthesia, nor caused any bleeding. The part of the elastic band that was protruding out of the gingiva was covered with plaque, imparting a yellowish colour and rough surface to otherwise blue and smooth elastic. Although the patient had noticed a yellowish “growth” on her gums, but since it was symptom-free, she did not report to a dentist.

The presence of foreign bodies in the gingiva may result in inflammatory response in surrounding tissues and subsequent loss of attachment apparatus, as reported in various case reports [5, 6, 8–10, 13]. This inflammation is independent of the degree of plaque colonization [14]. The condition may initially remain asymptomatic but usually presents clinically as pain, oedema, mobility, and/or pus discharge from the sulcus [6, 9, 13]. Becker and Neronov [5] reported abscess formation due to an elastic separator embedded in interdental space that healed completely after removal of elastic and periodontal curettage, leaving only mild alveolar bone loss. They emphasized the importance of appropriate imaging for diagnosis of such cases, which otherwise may remain unnoticed and continue periodontal destruction [5]. For effective and predictable management of such cases, early diagnosis of the condition is essential. In case the separator is missing at the time of banding, the patient should be asked about it. If the patient is unaware, then the area should be explored clinically and radiographically before banding. It is recommended that radiopaque and brightly coloured material be used for making orthodontic elastics, separators, and ligature bands to easily identify them on radiographs. Since clinical features are nonspecific and radiographs often fail to reveal the elastic bands, although metallic foreign bodies can easily be traced, a detailed history of previous dental treatments is of utmost importance. Depending on severity of the case, various treatment options may be tried. The area needs to be debrided of all the foreign bodies, granulation tissues, and calculus. The affected teeth may sometimes require splinting before surgical intervention. It may be required to raise a full thickness flap as in the case of Nettem et al. [6]. They reported a case of elastic band embedded between mandibular first and second molars that resulted in a deep pocket and abscess formation in otherwise healthy dentition of a 20-year-old female. They performed incision and drainage followed by raising a flap for retrieval of elastic. The area healed completely 1 week after surgery [6]. In severe cases, interdisciplinary approach may be required as reported by Al-Qutub [9]. He described the surgical management of a maxillary central incisor with grade III mobility, resulting from severe bone loss due to creeping of elastic placed for closure of midline diastema in a 9-year-old female child. After splinting, a full thickness flap was reflected to retrieve the elastic, followed by complete debridement, bone grafting, and placement of a resorbable membrane. Patient later required orthodontic intrusion after the tooth was found to be stable 6 months postoperatively. In most of the reported cases, antibiotic and analgesics were also prescribed to control the infection and pain. Specialized individual oral home care and regular monitoring of these areas are also important to prevent further breakdown of the periodontal attachment.

Fortunately, in this case, no significant periodontal destruction had occurred. Apparently, fair maintenance of oral hygiene in the affected area kept inflammation only subclinical. Still, the negligence in this case cannot be ignored, as it might have resulted in more severe conditions, jeopardizing the survival of teeth as reported in previous literature.

## 4. Conclusion

Elastic bands are commonly used for various purposes in orthodontics. It is advisable to do a thorough examination particularly in the interproximal areas for any residual material left at the completion of orthodontic treatment. Any area with periodontal destruction and history of orthodontic treatment should be inspected for the presence of foreign bodies. If diagnosed early, bone loss can be arrested and may even be regenerated if anatomy of defect is favourable.
